# Agriculture, COVID‐19 and mental health: Does gender matter?

**DOI:** 10.1111/soru.12408

**Published:** 2022-11-01

**Authors:** Hannah Budge, Sally Shortall

**Affiliations:** ^1^ Centre for Rural Economy School of Natural and Environmental Sciences Newcastle University Newcastle upon Tyne UK; ^2^ Duke of Northumberland Chair of Rural Economy Centre for Rural Economy School of Natural and Environmental Sciences Newcastle University Newcastle upon Tyne UK

**Keywords:** COVID‐19, farms, men, mental health, women

## Abstract

Agriculture is one of the most precarious professions, being vulnerable to weather extremes and animal disease. As crises hit the agricultural sector, a growing awareness and concern for the mental wellbeing of farmers developed. Economic decline, climate change and culling animals all have a profound impact on affected farmers. To date, research has tended to focus on the farmer, typically a man, and not the farm family. This article considers the impact of Coronavirus (COVID‐19) on men and women on farms. Using qualitative interviews and focus groups, the impact of the pandemic on men's and women's work and social life within the family is explored. We found a differential impact. For farmers, usually men, COVID‐19 was generally a positive experience both in terms of work and social life. For women, on the other hand, COVID‐19 was found to have a negative impact on their work and social life. While gender equality in agriculture persists, women's equality in the workplace has advanced. However, with the pandemic, women worked from home on the farm. They experienced a regression in gender equality with traditional expectations of responsibility for childcare and housework returning. In addition, their emotional and ‘mental’ labour increased. We conclude that in the future, the mental health of men and women on farms needs to be considered when crises occur. Crises impact the farm family and different members of the family in dissimilar ways.

## INTRODUCTION

Agriculture is a precarious industry, affected by weather extremes and animal disease. As crises hit over recent decades across the globe, concern for the mental wellbeing of farmers developed. In general, research has focused on the ‘farmer’, typically the man on the farm, and not women or other adults on the farm (for an exception, see Meyer & Lobao, 2003). Studies found that men's high levels of social isolation, masculine hegemony and a reluctance to engage with health services all impact men's mental wellbeing during crises (Henning‐Smith et al., 2022). It is a well‐established fact that over 90% of farms are family farms and rely on unpaid family labour (Graeub et al., 2016); hence, it is likely that crises, economic, climate or animal‐related, are likely to impact the family.

Agriculture is a notoriously unequal industry with men typically inheriting land and few women farming in their own right (Shortall et al , 2020). Research has consistently shown that even when women ‘marry in’, they make significant contributions to farm decisions and to diversifying income streams through their off‐farm employment (Shortall et al., 2022, 2017), Despite limited progress in gender equality in agriculture, there has been considerable progress in women's labour market equality in the global North (Fagan & Rubery, 2018; Guerrina & Masselot, 2018). Despite this growing equality in paid work, there has been less equal division of domestic and caring responsibilities (European Institute for Gender Equality, 2020). Research has shown that women on farms who have off‐farm work continue to be involved in farm work and have primary responsibility for domestic and care work (Sachs et al., 2021).

In this article, we consider the implication of COVID‐19 on men and women on farms by using a case study of Scotland. Farming is often seen as an isolated industry, and increasingly so over the years. During the pandemic, agricultural shows were cancelled, and marts/sales of cattle moved online, eliminating key social hubs for farmers. At the same time, household and caring responsibilities increased, as university students returned home, and younger children were home‐schooled. This article specifically focusses on the implications of COVID‐19 on men's social integration and mental wellbeing and the impact of increased care work on women during the pandemic and the implications on their mental wellbeing. Combining two studies, we examine the impact of these elements of the pandemic on men and women on farms.

We begin by reviewing some previous literature that examined the impact of farming crises on men's mental wellbeing. Then we turn to review some of the literature that has examined the impact of COVID‐19 on women. Next, our methodology is explained, which combines two datasets of qualitative interviews and focus groups. Then we turn to our analysis. We found that men were positive about their work and social life during COVID‐19, while it was the opposite for women. Women were expected to absorb all of the additional domestic and care work created by the pandemic, and they experienced an increase in emotional and ‘mental’ work. This happened alongside their off‐farm work and farm work. We conclude, similar to Alston et al. (2018), that when workloads increase, they tend to be defined as extensions of women's responsibilities rather than work to be shared. We also conclude that when future crises occur, the impact on the mental wellbeing of all adults in the farm household needs to be considered and not just the male ‘farmer’.

### LITERATURE REVIEW

#### Farm crises and men's mental wellbeing

A number of crises have led to analyses of the effect on men, such as the US farm crisis in the 1980s, the drought in Australia over the past 20 years and the foot and mouth crisis in Europe but mainly in the UK in the 2000s. Today's studies of the impact of the crisis on mental wellbeing have predominately focussed on men's mental health. The US farm crisis of the 1980s led to economic hardship for many farms, particularly in the Midwest. There was a concern for farmer wellbeing, and studies of farm mental health followed. The majority of these focused on the impact of economic hardship on men's wellbeing (Hoyt et al., 1997; Swisher et al., 1998), although Meyer and Lobao (2003) also found that economic hardship equally impacted women's mental health. The drought in Australia had severe effects, with a spike in farmer suicide, linked to the uncertainty of when, or if, the drought would end and the need to cull herds because of a lack of water (Alston, 2012). In Australia, high levels of social isolation were seen to add to psychological distress, alongside increasing debt. In addition, a masculine hegemony leads to a culture of stoicism in the face of adversity and a reluctance to seek help (Alston, 2012; Bryant & Garnham, 2012). Following the foot and mouth outbreak, Olff et al. (2005) argued that the outcome of reduced incomes, social isolation and most importantly the horrific burning of carcasses on infected farms led to many farmers having symptoms of post‐traumatic stress disorder. The images were distressing for the general public, but far more profound for farmers, and presumably for all members of the family farm. Farmers were seen as reluctant to engage with mental health services, and many mentally leaned on veterinary surgeons coming to the farm (Peck et al., 2002). Farmers are generally seen as reluctant to seek help, with pride being a major factor (Roy et al., 2014).

The general view is that farmers are expected to be silent stoic figures in the face of crises, and this has profound impact on their mental wellbeing (Henning‐Smith et al., 2022). Many imaginative initiatives have emerged attempting to tailor health and mental health services to men of farms. For example, the Lincolnshire Rural Support Network provides health screenings at marts and case‐work services at people's homes where they can discuss any health, family and financial issues (Lincolnshire Rural Support Network, 2022) This organisation was set up by a farmer's daughter after he completed suicide. In Scotland, this includes an ongoing survey created for and by farmers, with hopes to follow the New Zealand ‘Farmstrong’ model to create a wellbeing programme for Scottish farmers, working with existing charities and organisations (Davidson, 2022). The predominant focus of these provisions is on men. The mental health of farmers was highlighted in the Royal Agriculture Benevolent Institution (RABI) ‘Big Farmer Survey’ conducted in 2021 in England and Wales. The results found that 36% of participants were possibly or probably depressed (RABI, 2021), with 58% of women suffering from mild, moderate or severe anxiety.

#### COVID‐19, the family and implications for women

COVID‐19 had profound impacts on home life. During the initial lockdowns in 2020, there was a swift change to how we worked, with a general move to homeworking. This had many impacts on people's daily lives, including establishing makeshift offices in homes, negotiations over Internet usage and home‐schooling of young children and for some households, an influx of family members who may usually just be around during holiday periods. The press and research quickly found that this change had a variety of impacts on household members including mental health issues, conflict and an increase in domestic violence (Maria, 2021). The negative impacts affected everyone but they were mainly felt by women, who acquired the bulk of the responsibility for home‐schooling and domestic work (Budge & Shortall, 2022; Daly, 2021; Maria, 2021; Vilcu et al., 2021). Feminist scholars have long noted that the inequality in domestic and care responsibilities are significant barriers to achieving gender equality (European Institute for Gender Equality, 2020; Fagan & Rubery, 2018). Sociologists have identified women's responsibility for caring roles as a persistent social pattern (Shortall & Hansda, 2020). Caring roles tend to be formal, provided by the state or informal, and these responsibilities for children, elder care and the family predominately fall to women. Scholars have noted that this was the traditional role when women worked full‐time in the home (Lewis, 1992). However, as women have attained greater equality in the workplace, their responsibility for caring roles haven not equally diminished. This has led to what is often is called the ‘double burden’ of women's workload (Delphy & Lenard, 1992). Women often make decisions about their participation in the labour market based on these responsibilities (Daly, 2021). It is no surprise then that in terms of the pandemic, women were broadly expected to manage the sudden increase in childcare and caring responsibilities. This included looking after children, older relatives and general unpaid and often unseen care work (Vilcu et al., 2021). One implication of this was that some women struggled with home working throughout the lockdown. This was now combined with the additional responsibilities of caring, home‐schooling, domestic duties and an increased emotional burden (Daly, 2021).

A study during the pandemic found that women were more likely to spend time on home‐schooling and care work than men during the lockdowns and that they were more likely to reduce their overall working hours to do this unpaid care work (Xue & McMunn, 2021). Feminist scholars have noted that when there are economic downturns or fiscal austerity, gender equality loses momentum (Fagan & Rubery, 2018). Similar concerns arose during the pandemic. Women's step back from the workforce has implications for their potential future earnings, which could have a domino effect on the gender pay gap, effectively rescinding the progress made in the past few decades (Andrew et al., 2020).

### METHODOLOGY

This research combined two different studies. One study is the Economic Social Research Council (ESRC)‐funded study that wants to understand the position of equality for women in agriculture in the Scottish islands and two Atlantic Canadian islands and comprised 31 qualitative interviews. These were undertaken with men and women on farms, to better understand gender equality. Of these, 21 were conducted in Scotland and therefore included in this dataset, with those conducted in the Canadian islands being excluded. The second study was a rapid response to the Scottish Government that wanted to understand how COVID‐19 was affecting women on Scottish farms. This involved two focus groups, one of which was a 3‐h extended focus group with eight women. The other focus group involved two women and was conducted over a 2‐h period. In total, the research draws on 21 qualitative interviews and the views of 10 participants in focus groups. The ESRC‐funded research was conducted during COVID‐19. Although the purpose of neither data collection was about the mental health implications of the UK lockdowns in the agriculture industry, it became an evident theme throughout the interviews and focus groups and warranted further examination and analysis. The result of the analysis is this article. Both researchers are from a farm background. One researcher worked from the farm during the lockdown and had a real appreciation of how family dynamics changed during COVID‐19.

The combination of these datasets resulted in a broad geographical range, which included those who were within the borders of Scotland and the islands. The participants were a mixture of those who were directly involved in the agriculture industry, that is, full‐time farmers/crofters^1^; people whose partners were crofters or farmers; people who worked in connection with the industry and those who were part‐time crofters. This provided a range of experiences of how COVID‐19 affected those involved in the industry. The sample is reflective of the diverse roles that collectively make up the Scottish agriculture industry. As well documented, farming and crofting households are largely embedded within the holding, and although there is always a ‘main’ farmer, they usually heavily rely on family labour (Shortall et al., 2017; Budge and Shortall, 2020). Therefore, when considering the impact of COVID‐19 on people in agriculture, it was important to consider all those who make up the household rather than purely focus on the lead/main farmer themselves (Tickamyer, 2020). What we mean when we refer to the lead farmer is the person whose main occupation is running the family farm. This person tends to be the registered agriculture holder. The research itself took place in 2020 and 2021, during the COVID‐19 lockdowns and when various restrictions were in place in Scotland. All focus groups and interviews took place on zoom.

### FINDINGS

A key difference that emerged in the analysis was between those who were the ‘lead’ farmer and those who were not. The impact of the pandemic on their mental health was very different.

In general, for the lead farmer, the experience of the pandemic was positive. The number of women lead farmers in the sample was five, with the rest either running the holdings with partners or who had married into the farm and helped on the holding but did not take part in the management side of the business. Their experiences were mixed, where all the male participants were lead farmers and all had similar experiences. Some women found it a positive experience, whereas others did not. It is to this analysis that we now turn.

#### Lead farmer

Marts and agricultural shows were cancelled during COVID‐19, and there was an expectation that this would lead to increased social isolation for farmers. However, this was not the case. Farmers interviewed, in the main, felt *more* socially integrated. Farmers were positive about children returning home during COVID‐19 and helping on the holding. It also meant more people were in the household throughout the day rather than the farmer being by themself. The fact that events were cancelled, meant there was more time to be spent on the holding, with family members spending time working on needed jobs. While Brexit was initially expected to lead to economic hardship and lower prices, the opposite occurred. The price of meat increased, and farming buoyed during COVID‐19.

Farmers were very pleased about having family return to the farm and having a group of ‘willing helpers.’ Farmers were also positive about social events being cancelled. It meant there was a focus on the holdings, and jobs such as fencing and generally tidying up were prioritised.

One farmer highlighted the lack of distractions meant that they could focus on their holding rather than on their tourist diversification business:

It's been really good. This sounds bad to say it also not having to deal with tourists and stuff. In our bothy^2^ even though obviously it's a fall in income, we did get some government money to replace that, which means I've really been able to focus on the farm properly instead of sort of, you know flipping between lots of different jobs.

The influx of family members and lack of distractions meant that more jobs could be completed on the holdings. The lockdowns were seen as positive for some farmers and crofters who completed outstanding tasks.

Another positive feature of the pandemic that emerged was for farmers and crofters who farm or croft part‐time. The initial lockdown occurred in March 2020, and for many, this was lambing time. By being able to work from home, people could be more flexible during that busy period as depicted by one man:

I've been working from home for the last year, which has been excellent for the croft… (referring to lockdown), it just came at the right time as well with lambing last year.

Another woman referred to her son who now runs their family croft and that they could use working from home to their advantage:

Michael usually took off holidays for a couple of weeks so…he was actually working from home, so if there was an emergency he could just pop out and in again.

This demonstrates the advantages for farmers that the lockdowns brought with the shift to home working. The family were at home and on hand.

The initial lockdown came at a busy period of the year. Farmers and crofters still had to attend to livestock. Classed as essential workers, they still had a job to focus on unlike those who were furloughed. For some, this is what helped them through the pandemic, it meant they still had a purpose. Those in remote rural areas in this sample, where there were very low rates of COVID‐19, said they did not worry about becoming ill because they were less likely to be exposed to the virus. Combined with the ability to go outside and carry on with their farming and crofting work, meant that for many their mental health was not affected:

I think it was…you just kind of got on with it, you had to feed your animals, you had to get the land ready and such like that, so we were very lucky that way it's not impacted on our mental health because we've been active and had plenty to occupy ourselves with.

A further unexpected positive impact was the rise in lamb and meat prices. During a time of uncertainty, with Brexit and the closure of the hospitality sector due to COVID‐19, this increase in prices meant that it mitigated the loss of earnings for some who did agriculture contract work. As expressed by this man, a full‐time crofter:

But now at this stage, then, we the lockdown, rather as folk eating out then there would be the new generation now that would actually have started to learn to cook and realise they enjoy it, so as far as we would be concerned at both beef and lamb prices is booming and that is solely at the back of demand. And demand within Europe as well. And this year we the lead up to Brexit then it should have been flat and dismal, but as long as none of us gets it we the second wave then the sooner, the longer this keeps on the better! From agriculture point of view.

The price of meat at the time was at a high, demonstrated by this male farmer:

So there's been a real switch back to supporting local and supporting British and I don't think…would we have seen the beef price rise from what about £3.40 a kilo up to £3.80? Somebody said…did it from being what was it a 10‐year low to a 5‐year high or a 5‐year low to a 10‐year high in a matter of months or weeks.

In terms of women, our findings were mixed. One participant, a woman who is the lead crofter, expressed the benefits of having family members return to the holding:

Very positively as we touched on earlier, one of my daughters and her boyfriend came back here just before lockdown last March and I had a list that long in my head of projects that I would do if I had a willing team of helpers. The weather was wonderful…So…as the days progressed and as lockdown continued and one lockdown led to another level of lockdown, we just remained here, and everybody was working from home doing their various things and our day was planned around doing whatever work we had to do and then continuing with the croft projects.

This is very similar to the general theme for lead farmers. Another woman farmer however reported a completely different scenario. When her husband had to work from home, she was expected to undertake additional household duties:

F*****g ewes, sorry for swearing. Eight hundred ewes, calves, cows and like, one example was I put a ready meal in the oven that nobody wanted to eat because they couldn't decide what they wanted to have, and then they wanted Yorkshire puddings with it. So in‐between the 10 min the Yorkshire puddings had to cook I had to go f*****g well catch two ewes with mastitis, honest to the lord. And they're like looking at me waiting to be fed, I wanted to stab them… Regressed to a women's place that all the progress I thought I had made in my life seemed to have slipped away. I was the responsible functioning adult that had to cook and do the shopping and lamb ewes and calve cows.

This woman saw her position of equality reversing. Her husband did not see that her full‐time job was continuing and instead expected her to fulfil traditional roles of housekeeping and cooking. Another woman who farms in partnership with her husband reported that she assumed full responsibility for home‐schooling, which she found very stressful:

I gave up my career to work with family on the farm. It was the right thing to do at the time–it was right for the kids–for their mum to be there to take them to school, to feed them, to pick them up etc. as it was expected of me as their mum–what wasn't expected or even questioned was for my husband to do this–for him to give up some of this time, for him to change his routine to fit around the kids and I am very resentful about this. The farm has definitely turned into home‐schooling. I think from the start I just said that it's not something I can do…I send my kids to school for a reason, there are teachers for a reason and I am not equipped to deal with that with them at all. I've tried to incorporate them (into the farm) and obviously because they're home more now so the job role changes.

This woman also talked about her parents‐in‐law expecting her to do more formal home‐schooling with her children, which she resented as she felt ill‐equipped to do so. The same expectation was not made of her husband.

While the RABI Big Farmer Survey reported mental health and wellbeing issues in the agricultural industry, our research did not generally find this to be the case for lead farmers. Social contact increased as children returned home, and spouses/partners worked from home. The sector buoyed with increased profits during the pandemic. The only exception was for some of the women farmers who found that the traditional expectations of housework and childcare increased their workload. We now turn to women who work off the farm and who returned to the holding to work as required by the lockdown.

#### Women who work on‐ and off‐farm

Women throughout the UK and elsewhere during the pandemic were largely expected to take on the increased domestic and home‐schooling tasks. We did not anticipate that it would be different within the agriculture sector. Here, the experience of the lockdown was very different to that of the lead farmer. For instance, for one participant who helps on their husband's croft, as well as leading their diversification business being closed during the lockdowns, it disrupted their routine and their focus.

My normal routine was non‐existent because no, our business was shut. There weren't as many people on the island, and you know it's just that sort of social thing, so I really struggled mentally with the whole thing because it's just like somebody pulled the rug from under your feet, those things that you normally to do like see family, you might you know it's just was really hard, but I think my husband and son, they just, life just continued because there was something what they normally do because obviously still have to you know, the animals and all the work on the croft so that didn't stop you know that just carried on.

This quote demonstrates the differential impact of the pandemic on different family members. While farming continued, those who had on farm‐related tourist businesses were heavily impacted, for instance, loss of sales and bookings as demonstrated by another female farmer:

I did it, farm diversification, year before last and to build any eco bothy on our farm. That was 50% funded by the EU. So that was one of the other things that Brexit made me focus on, was getting trying to spread the risk of it. And then COVID happened. So had lots of tourist bookings and then had to cancel them.

Women were often resentful that their partners did not fully appreciate how much their life had changed during lockdowns, in comparison to his:

I keep arguing to Fred that his life has carried on pretty much as normal, whereas mine changed, completely changed on its head because I took on the home‐ schooling, and because I was home he was able to go out and do far more than what he would have been otherwise just by himself, whereas I was home and just doing the kids. My job changed but the farm didn't really change, it carried on as normal.

The same participant commented that since they were working from home, their partner additionally expected them to help out more. Their makeshift office was positioned above the lambing fields, which meant they had to juggle several roles at a time.

It coincided with the start of lambing I was doing IACS^3^ forms from home, trying to home‐school, and Fred would then say keep an eye on those ewes they're going to lamb today. So I was focusing on mainly being in the office and then he would just be there to get on with it. So yeah it was probably the most involved I've ever been because I was at home because of lockdown yeah.

Interviewer: And how did you find that having to juggle all those different roles?

Really hard. [Laughter] Really hard. I would have to leave a call or a Teams meeting to run out and catch an ewe so the computer looked out onto the lambing park and yeah the kids would come in and say that ewe is lambing so I'd have to leave the call and go and…the kids are too wee to handle that themselves. So…and my husband does lots of off‐farm work, so he works at the mart, and so he's quite often not home, so we muddled along is how you can describe that.

This demonstrates the frustrations that built up during that period, how women in this sample had to juggle many roles within the household itself and assume all of the additional labour generated by COVID‐19. One woman described the burden of home‐schooling:

I have been spending more time doing office stuff, and this is going to sound like a broken record, obviously having to manage the childcare and home‐schooling. Because effectively you don't have the childcare provided by the school if you like, and also afterschool clubs, which you know we were involved in up until 5 o'clock or half past four in the evening. So you're suddenly trying to juggle, still being involved in meetings and trying to do the farm business's paperwork and everything else, trying to provide some home‐schooling routine for a child. I mean I'm lucky that I've only got one.

This woman has assumed all of the responsibility for home‐schooling. She also discussed how her husband had decided he needed to be outside ‘12 h a day’ and took no responsibility for the additional work generated by the pandemic. She said that this lack of involvement had put ‘extra pressure’ on their relationship.

One woman with a senior position in an organisation reported the increased amount of mental labour and how difficult she found that:

Mentally, my biggest issue has been headspace and it's going to sound, and I don't mean to sound kind of poor me, the cooking, I don't mind but the having to make a decision every single day for what we are eating, what we are buying at the shops, what time the kids are getting out of bed, what we do during the day is overwhelming, on top of work. That's been the hardest thing for me…I totally lost it a few weeks ago when I was trying to say to people that I need others to step up. And there was a complete reliance on me to decide everything about everything for them because that was easiest for them.

There was general agreement in the focus group that all of the mental and emotional labour created by the pandemic fell to them as women. The woman interviewed above went on to explain how her husband did not take any responsibility for the additional work created by the pandemic. As well as the farm, he does maintenance work on other farms:

So he jumped in the digger and he was off, and I had to tell to him stop working because I can't do this. You know I can't, I can't be working my days and then have you coming home at half six, seven at night asking what's for tea, and the kids coming through looking for tea as well so I've had to ask him to cut back his work, just to try and be the back‐up, he won't be doing any home‐schooling,

There was a general acceptance that the burden of home‐schooling would fall to women. In no case did we find a man who had assumed responsibility for home‐schooling.

Young adult children often had internships or graduate jobs withdrawn because of COVID‐19. This led to an increase in ‘emotional’ labour:

My daughter was due to do a summer's internship, that's fallen through, so she's, she's at home until at least September with no job for summer. Her and her girlfriend were supposed to be away travelling just now, her girlfriend is in France so I'm having to deal with that whole emotional turmoil that she's in as well. My son was due to start learning to drive, he can't even apply for a provisional driving license. His whole life revolved around his mates and football. So yeah, so they are needing a hell of a lot of emotional support just now, and unsurprisingly that falls to me.

The additional work created by the pandemic fell to women. It was seen as an extension of women's work, of household tasks and of women's nurturing role, and it was not necessarily even recognised as work. This created increased pressure on women with consequent challenges to mental wellbeing.

## CONCLUSION

This article examined the mental health impact of COVID‐19 on men and women on farms. It was not a farming crisis as such, in that it did not only impact farm families in the same way as the US farm crisis, drought in Australia or foot and mouth disease. It did however increase social isolation as agricultural shows were cancelled and marts closed. We did not however find that this had a negative impact on the wellbeing of men who farmed. Men reported more social contact as partners home‐worked in the farmhouse and adult children returned to the farm from university. They had more company, and people willing to help with jobs on the farm. Many reported catching up on jobs that were not immediate priorities. It was not a time of economic hardship for most, as prices and profits increased. The results for women who were the main farmer were mixed. For some it was positive, for others it was not, as responsibility for the additional housework and childcare/home‐schooling fell to them. For women who were working from home in the farmhouse, a more negative picture emerged. Women reported the mental difficulties of this additional work. They also described the mental labour involved in the additional decisions that had to be made daily and the emotional support needed by older children. Women felt that their husbands used the farm as an escape and worked very long days to avoid the need to engage with the additional work generated by COVID‐19.

Agriculture is not a gender‐equal occupation. Women on farms have advanced their position of equality by working off the farm. This article demonstrates that equality cannot be achieved until the domestic and care responsibilities are shared between men and women. This has to be the future focus of any drive for gender equality. We also conclude that studies of mental health need to focus on the impact of agricultural crises on both men and women on farms and not just the person seen as the ‘farmer’. Our research found the pandemic had very different outcomes for men and women on farms, and there is no reason to assume this might not also be the case for agricultural crises.

Our study has a number of limitations. Neither dataset was designed to assess the impact of COVID‐19 on the mental health of men and women; we analysed it because it was such a recurrent theme, both positive and negative. Our studies only assessed the mental health implications of the crises for adult men and women on the farm. We do not know how the pandemic impacted the mental health of children and young people on farms or even if it impacted their safety as they were cared for on the farm during lockdowns. These questions require further future analysis.

## FUNDING INFORMATION

Economic and Social Research Council; Scottish Government

## CONFLICT OF INTEREST

The authors declare that there is no conflict of interest that could be perceived as prejudicing the impartiality of the research reported.

## Data Availability

Research data are not shared.

## ENDNOTES

1 Crofting is a land tenure system that is specific to Scotland, where small areas of land are usually rented from landowners. These can range in size from just half an acre to 50 acres.

2 A bothy is a small building that was traditionally used for farm workers on a holding. It is now popular for these to be converted into accommodation for tourists.

3 Integrated Administration and Control System is a database system that controls and administrates direct payments, for instance agriculture‐environmental schemes and rural development payments.
